# The First Dinosaur from Washington State and a Review of Pacific Coast Dinosaurs from North America

**DOI:** 10.1371/journal.pone.0127792

**Published:** 2015-05-20

**Authors:** Brandon R. Peecook, Christian A. Sidor

**Affiliations:** 1 Department of Biology, University of Washington, Seattle, Washington, United States of America; 2 Burke Museum, University of Washington, Seattle, Washington, United States of America; Royal Ontario Museum, CANADA

## Abstract

We describe the first diagnostic dinosaur fossil from Washington State. The specimen, which consists of a proximal left femur, was recovered from the shallow marine rocks of the Upper Cretaceous (Campanian) Cedar District Formation (Nanaimo Group) and is interpreted as pertaining to a large theropod on the basis of its hollow medullary cavity and proximally placed fourth trochanter. The Washington theropod represents one of the northernmost occurrences of a Mesozoic dinosaur on the west coast of the United States and one of only a handful from the Pacific coast of Laramidia during the Cretaceous. Its isolated nature and preservation in marine rocks suggest that the element was washed in from a nearby fluvial system. If the femur pertains to a tyrannosauroid, which seems likely given its size and the widespread occurrence of the group across Laramidia during Late Cretaceous times, then it would represent an earlier occurrence of large body size than previously recognized (complete femur length estimated at 1.2 meters). Uncertainty surrounding the latitude of deposition of the Nanaimo Group (i.e., the Baja-British Columbia hypothesis) precludes assigning the Washington theropod to either of the putative northern or southern biogeographic provinces of Laramidia.

## Introduction

**Fig 1:**
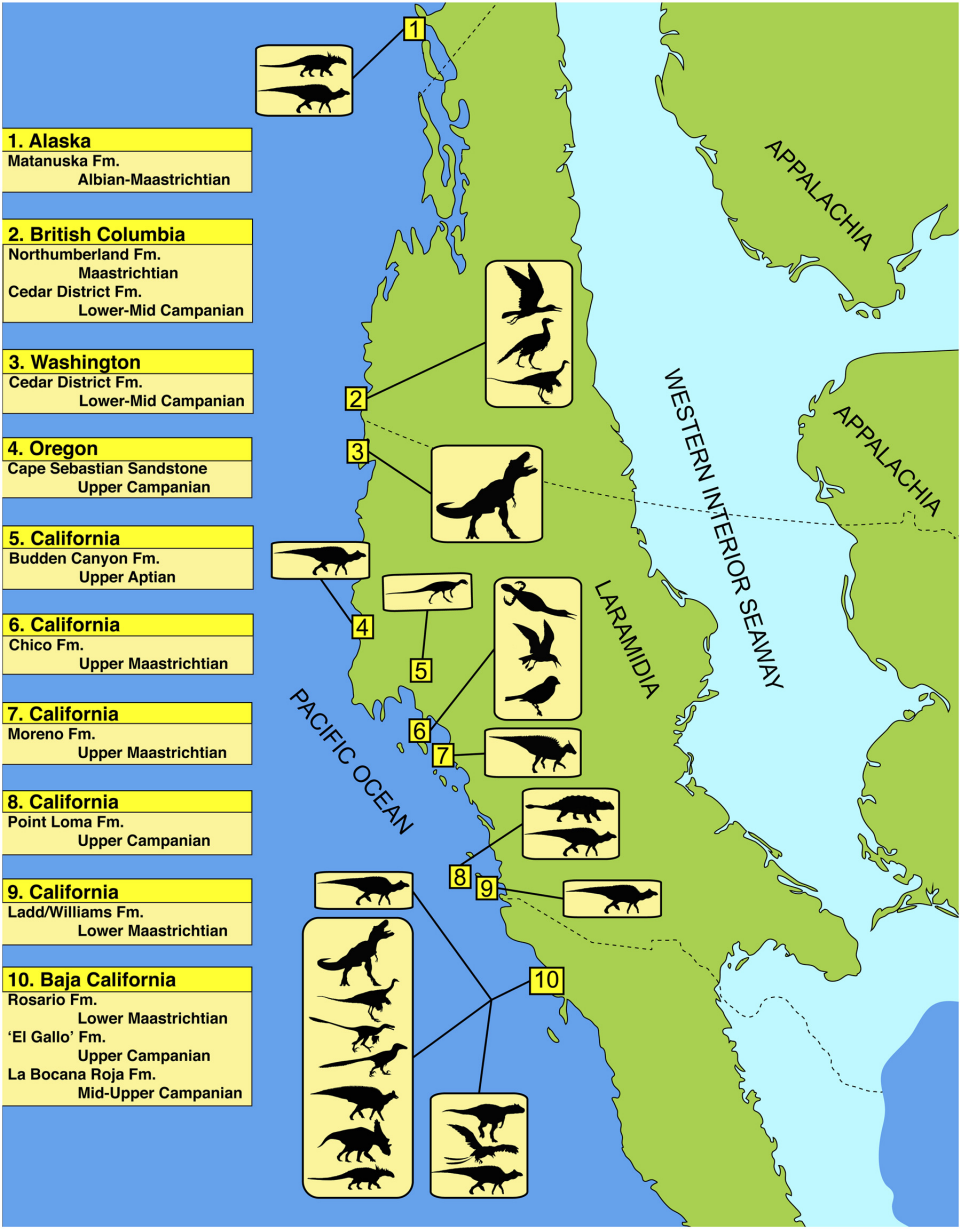
Geographic overview of Late Cretaceous dinosaur assemblages from the west coast of Laramidia. The specimen described here was collected from Campanian rocks at Sucia Island (Washington State), but these strata were probably deposited at a more southern latitude, perhaps as far south as the ‘El Gallo’ and La Bocana Roja formations of Baja California, Mexico. See Discussion for overview of specific taxa from each formation. Dinosaur assemblage data were gathered from the following sources: Matanuska Formation [13–15], Northumberland Formation [16, 17], Cedar District Formation [16], Cape Sebastian Sandstone [2, 18], Chico Formation [19, 20], Budden Canyon Formation [21], Moreno Formation [22, 23], Point Loma Formation [24–26], Ladd Formation [2], Rosario Formation [6], ‘El Gallo’ Formation [6, 22, 27–32], La Bocana Roja Formation [33, 34]. Silhouettes modified from images available on Wikimedia Commons.

The Late Cretaceous was a time of remarkably high dinosaur diversity worldwide, but especially so in North America [1, 2]. During the Cretaceous, a shallow inland sea separated North America into two large landmasses, Appalachia to the east and Laramidia to the west (Fig 1), with the bulk of dinosaurs recorded from non-marine rocks that were deposited along the eastern margin of Laramidia [3]. The dinosaurs of Laramidia have been considered to form two biogeographic provinces, a northern one represented by the rich fossil record of Montana and Alberta, and a more poorly understood southern province including localities in Utah, New Mexico, Texas, and Coahuila [3–6], but the reality of these provinces has been challenged [7]. In addition, several important dinosaur finds have been made in the marine rocks that represent deposition within the Western Interior Seaway itself (e.g., Bear Paw Shale Formation, Niobrara Chalk Formation, etc. [8–12]).

Compared to the areas in and around the Western Interior Seaway, relatively little is known about vertebrate assemblages, including dinosaurs, from the Pacific coast of Laramidia. Factors such as its active tectonic margin and high degree of human development have likely conspired to diminish the opportunity for paleontologists to make comparable discoveries. Most of the dinosaurian material from the Pacific coast comes from two formations in Baja California, México and pertains to hadrosaurid, ceratopsian, and ankylosaurian ornithischians, as well as many lineages of theropod saurischians, including tyrannosaurids, ornithomimids, dromaeosaurids, troodontids, and an enantiornithine bird [22, 27–31, 33–35]. Isolated skeletons and bones of ankylosaurian and hadrosaurian dinosaurs have been discovered in coastal or marine Cretaceous rocks in Oregon and California [2, 19, 20, 24, 26, 36], including the nodosaurid ankylosaur, *Aletopelta coombsi* [25] and the saurolophine hadrosaurid, *Augustynolophus morrisi* [23]. Enantiornithine and ornithurine birds are known from coastal British Columbia [17]. A single partial skull of the ankylosaur *Edmontonia* and postcranial elements of a hadrosaur were described from the marine Matanuska Formation of south-central Alaska [13–15]. Here we describe the first dinosaur from Washington State and add a new geographic record for Campanian theropods from the west coast of North America.

## Methods

The fossil described herein, UWBM 95770, is housed in the paleontology collections at the University of Washington Burke Museum (Seattle, Washington). It was collected under Sucia Island State Park research permit #080603. Locality information is available to qualified researchers. The authors gathered comparative measurement data at the Royal Tyrrell Museum of Palaeontology (TMP; Drumheller, Alberta, Canada) with permission of the relevant curator. The relevant specimens measured are listed in Table 1 (TMP 1981.000.0028, TMP 1986.205.0001, TMP 2001.036.0001, TMP 1982.013.0030, TMP 1986.205.0001, TMP 1981.010.0001, TMP 1994.012.0602, TMP 1993.115.0001, TMP 1993.115.0001, TMP 1967.014.0038, TMP 1994.036.0500, TMP 1995.038.0001, TMP 1979.020.0001, TMP 2001.000.0014, TMP 2004.003.0007, TMP 2000.050.0006).

**Table 1:** Linear measurements from selected theropod dinosaur femora.

Taxon	Specimen	Side	Proximal width	Length
*Tyrannosaurus rex*	TMP 1981.000.0028	left	280	1310
Theropoda indet.	UWBM 95770	left	214	1167*
*Albertosaurus sarcophagus*	TMP 1986.205.0001	right	130	1035
*Daspletosaurus torosus*	TMP 2001.036.0001	left	203	987
Tyrannosauridae indet.	TMP 1982.013.0030	left	211	985
*Albertosaurus sarcophagus*	TMP 1986.205.0001	left	140	980
*Albertosaurus sarcophagus*	TMP 1981.010.0001	left	166	928
*Gorgosaurus libratus*	TMP 1994.012.0602	right	179	915
*Sinraptor dongi*	TMP 1993.115.0001	left	142	855
*Sinraptor dongi*	TMP 1993.115.0001	right	145	850
?Tyrannosauridae	TMP 1967.014.0038	left	106	704
*Gorgosaurus libratus*	TMP 1994.036.0500	right	86	635
Dromaeosaur indet.	TMP 1995.038.0001	right	74	508
*Chirostentotes pergracilis*	TMP 1979.020.0001	right	51	297
*Saurornitholestes langstoni*	TMP 2001.000.0014	right	35	206
*Saurornitholestes langstoni*	TMP 2004.003.0007	right	31	203
*Avimimus portentosus*	TMP 2000.050.0006	left	20	177

All measurements in millimeters. Proximal width was measured from the junction between the neck and head of the femur transversely to the lateral surface of the element. Asterisk denotes estimated length based on a linear regression on the log-transformed data shown here.

## Geological Setting

Rocks of the Nanaimo Group crop out on Vancouver Island and the Gulf Islands of Canada as well as the nearby San Juan Islands of Washington State [37]. They range from Santonian to Maastrichtian, have been subdivided into 11 formations, and host a diverse invertebrate fossil record including ammonites, baculitids, and inoceramid bivalves upon which a regional biostratigraphy has been developed [38–43]. The Cedar District Formation, which lies near the middle of the Nanaimo Group, was interpreted at Sucia Island as representing a shallow marine shelf facies by Ward et al. [41]. Elsewhere the formation includes shales, turbidites, and conglomeratic units that can be difficult to correlate between outcrops scattered within the San Juan Islands and elsewhere [40].

At Sucia Island, the Cedar District Formation has been correlated to the upper part of the chron 33r interval and spans the *Hoplitoplacenticeras vancouverense* and *Baculites inornatus* range zones [41, 44]. Haggart et al. [45] considered the Cedar District Formation to be upper Campanian, whereas Ward et al. [41], who used an updated biostratigraphy, as well as magnetostratigraphic and preliminary strontium isotopic data, considered it lower middle Campanian (~80 Ma). The paleolatitude at which the Nanaimo Group was deposited is contentious, with advocates of the Baja—British Columbia hypothesis suggesting a paleolatitude of approximately 30° north (i.e., equivalent to Baja California today [44, 46, 47]). This hypothesis implies over 3000 km of northerly displacement between the site of original deposition and the present position of Sucia Island strata. Alternatively, the rocks of the Nanaimo Group have been interpreted as having been deposited much closer to their current location, or at a latitude corresponding to that of present day northern California [48, 49].

## Results

### Systematic Paleontology

Dinosauria Owen 1842 [50]

Theropoda Marsh 1881 [51]

Theropoda indet.

#### Referred specimen

University of Washington Burke Museum (UWBM) 95770, proximal portion of left femur.

#### Horizon and locality

Collected from a dark gray silty sandstone of the shallow marine Cedar District Formation, Nanaimo Group at Ev Henry Point, Sucia Island State Park, Washington (locality UWBM C1659). Based on invertebrate biostratigraphy and magnetostratigraphic correlation, Ward et al. [41] considered the Cedar District Formation to be early middle Campanian (~80 Ma) in age.

#### Diagnosis

**Fig 2:**
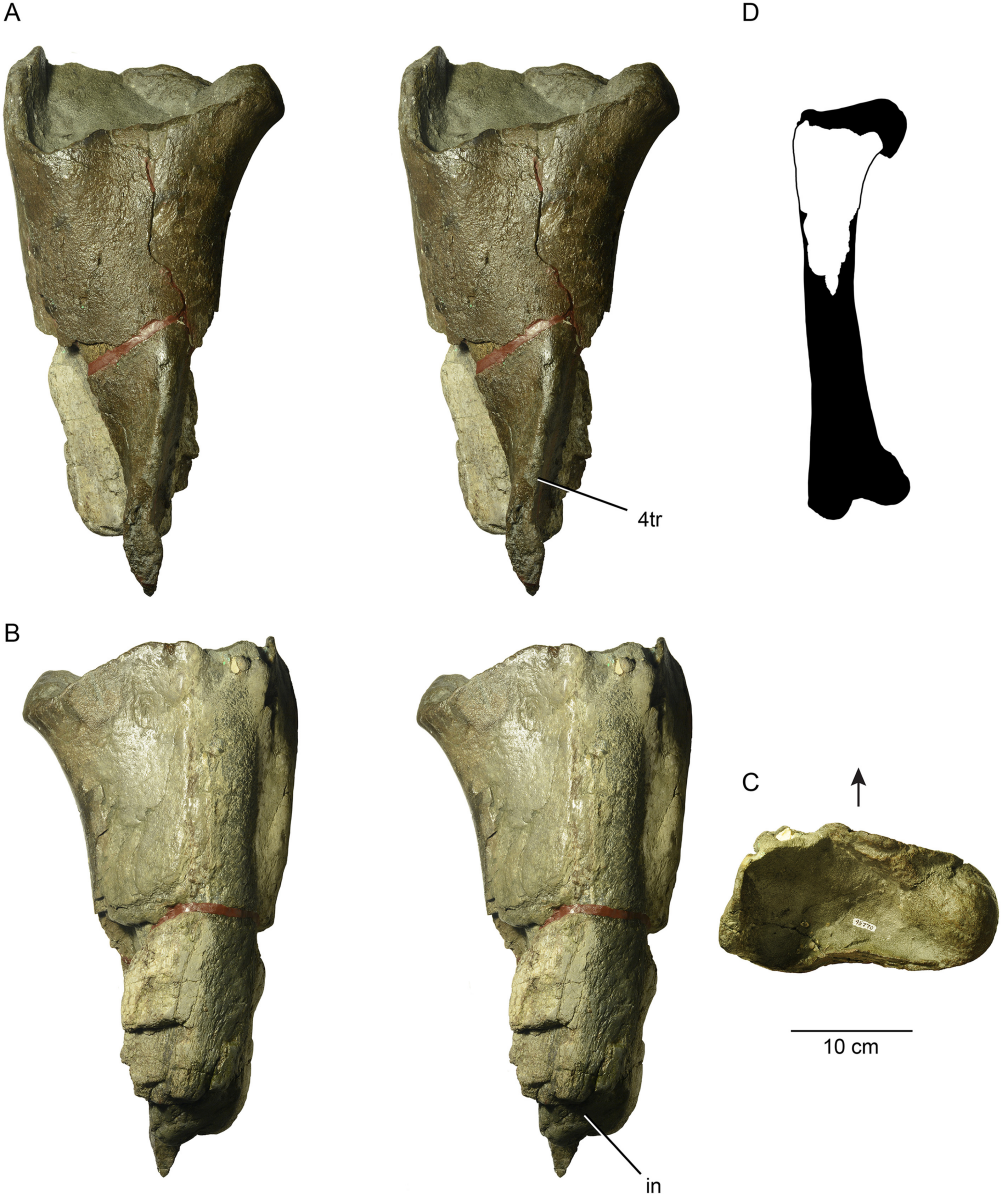
The proximal femur of a large theropod dinosaur from Washington State. Stereopairs of UWBM 95770 in posterior (A), anterior (B) view. Proximal view (C) of UWBM 95770, with arrow indicating anterior direction. Silhouette of complete theropod femur (D) based on the tyrannosaurid *Daspletosaurus torosus* (TMP 2001.36.01), with corresponding portion of UWBM 95770 highlighted. **Abbreviations**: 4tr, fourth trochanter; in, matrix infilling of hollow marrow cavity.

**Fig 3:**
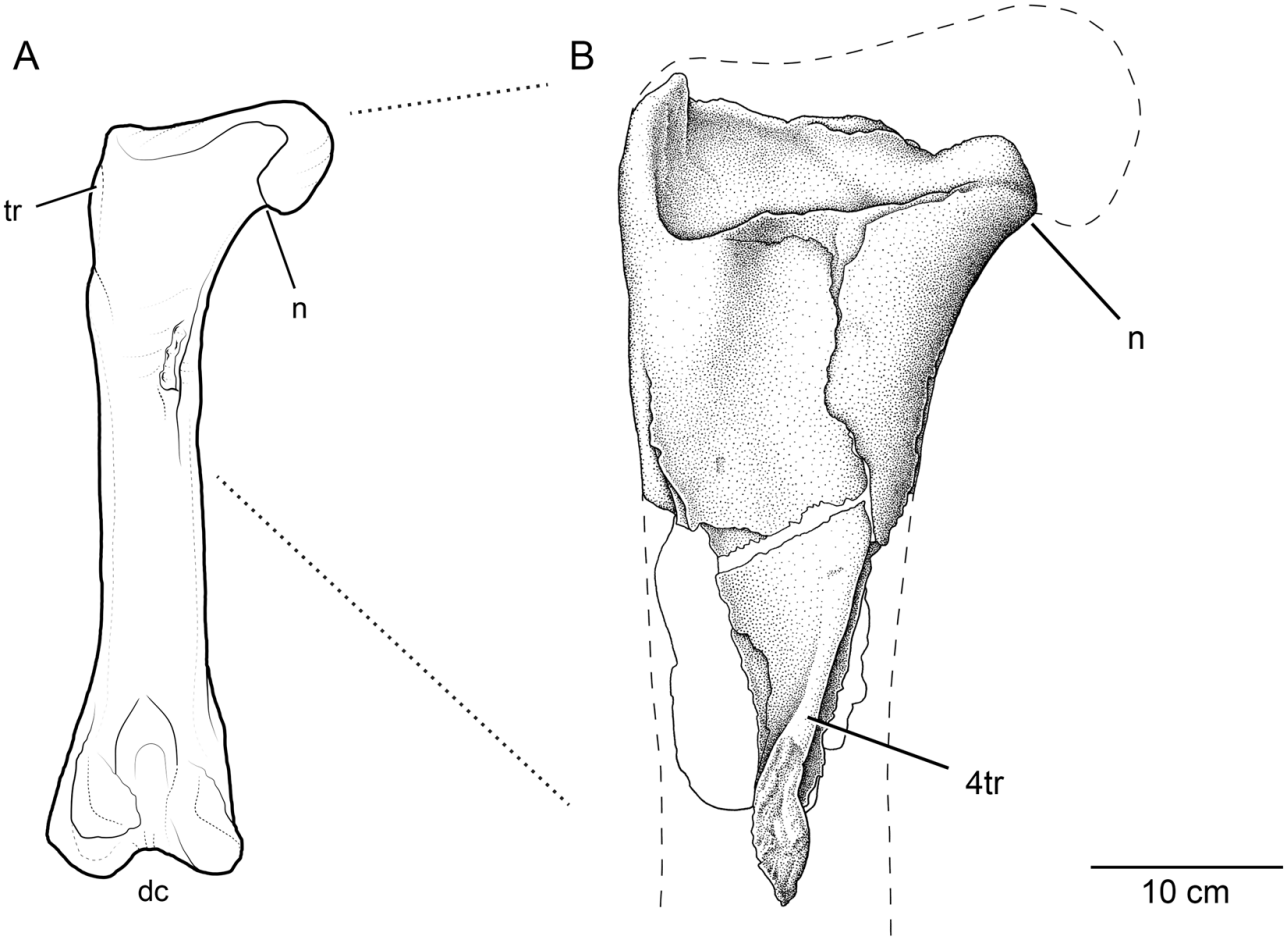
Comparison of theropod dinosaur femora in posterior view. Illustration of complete theropod femur (A), based on the tyrannosaurid *Daspletosaurus torosus* (TMP 2001.36.01; total length 987 mm) and (B) the Washington State theropod (UWBM 95770). **Abbreviations**: 4tr, fourth trochanter; dc, distal condyles; n, femoral neck; tr, area of greater trochanter (but this feature is better seen in anterior view).

UWBM 95770 can be identified as a theropod dinosaur based on its possession of a hollow medullary cavity and the relatively proximal position of the fourth trochanter (Figs 2 and 3).

### Description and Comparisons

We interpret UWBM 95770 as the proximal portion of a left femur. As preserved, the element measures 42.5 cm in length and 22.4 cm in maximum width, across what remains of the femoral head. At its preserved midlength, the element has a circumference of 40.6 cm and is notably wider medio-laterally (14.6 cm) than cranio-caudally (11.9 cm), although the latter measurement must be considered a minimum due to weathering. In posterior view (Figs 2A and 3B), the specimen is roughly triangular in shape, with a broad proximal region tapering towards the remnants of the high and elongate fourth trochanter. As a result of being exposed in an intertidal region, the anterior face of UWBM 95770 is deeply weathered to display the inner spongiosa (Fig 2B). In distal view, the cross-section of the in-filled medullary cavity measures (minimally) 6 cm wide and 3.5 cm anteroposteriorly.

Only the base of the femoral neck is preserved (Fig 3). Erosion appears to have particularly affected the dorsal and articular surfaces of the femur, possibly because these areas lacked a finished periosteal surface. In fact, fossil invertebrates, including the common bivalve *Crassatellites conradiana*, were found scattered inside the body of the proximal femur, suggesting that biological activity was at least partially responsible for this taphonomic loss. In proximal view (Fig 2C), the specimen has a relatively straight posterior margin and a broadly arched anterior outline. The proximal shape differs substantially from the proximal view of complete theropod femora (e.g., [52]), but the absence of the large lesser trochanter in UWBM 95770 must be kept in mind. In anterior view, the periosteal surface is generally complete and well preserved, except for the middle one-third, which is deeply eroded such that trabecular bone of the interior is visible. Erosion becomes more prominent distally, with the hollow interior of the bone represented by a dark gray siltstone matrix infilling. The area of the greater trochanter preserves compact bone of the periosteum, but it is difficult to determine if it represents the original external surface or a slightly deeper level.

The apices of the complete periosteal surfaces of the base of the femoral neck and the greater trochanter are in-line with one another across the long axis of the preserved cross-section. This morphology suggests that the missing femoral head would have been oriented directly medially, and not anteromedially as in non-tetanuran theropods [53, 54].

The posterior aspect of the femur is generally well preserved and features the most diagnostic aspect of its anatomy, a distinct fourth trochanter (Figs 2A and 3B). Proximally, the fourth trochanter is somewhat inset from the medial surface of the femoral shaft and runs slightly laterally across the shaft towards its distal termination. In cross-section, the proximal portion of the trochanter has a steep-sided medial surface and a relatively broad lateral surface that blends into the posterior face of the femoral shaft. Although the surrounding femoral shaft is incomplete, the distal portion of the fourth trochanter appears much more prominent and ridge-like. In addition, the proximal half of the trochanter has a smooth surface whereas the distal half is rugose, which suggests that this muscle attachment continued in cartilage in life. A similar condition is seen in *Daspletosaurus torosus* (TMP 2001.36.01), where the middle portion of the fourth trochanter is rugose and unfinished (Fig 3A).

When taken in combination with the presence of hollow bone, the anatomy of the fourth trochanter supports the identification of UWBM 95770 as a non-paravian (non-eumaniraptoran sensu [55]) theropod dinosaur (e.g., a ceratosaur, megalosauroid, allosauroid, or tyrannosauroid). A relatively proximal location of the fourth trochanter, typically within the proximal one-third of the femur, is diagnostic of theropods among Cretaceous dinosaurs [56], although this placement likely first evolved among Triassic dinosauriforms [57, 58]. In other large-bodied Cretaceous dinosaurs, such as hadrosaurids or ceratopsians, the fourth trochanter is placed near the femoral mid-shaft and takes the form of a triangular prominence or long low ridge, respectively [59–61]. Although the specimen is incomplete, measurement data demonstrate that UWBM 95770 preserves enough anatomy to indicate a theropod-like position of the fourth trochanter. It should be noted, however, that derived paravian theropods are characterized by a reduced fourth trochanter that can be nearly indistinguishable from the femoral shaft [55].

### Predicted Total Length of UWBM 95770

In order to compare UWBM 95770 more broadly with other large theropods, we used measurement data to estimate its total length when complete. Linear measurements were taken from theropod femora housed in the Royal Tyrrell Museum of Palaeontology (Table 1). Proximal width was measured from the junction between the neck and head of the femur transversely to the lateral surface of the element. Length was measured from the dorsal-most point of the proximal surface, usually above the point where the femoral neck and head meet, parallel to the femoral shaft to the distal-most prominence of the condyles.

We used R [62] to compute a logarithmic line of best fit between femur proximal transverse width and total length (r^2^ = 0.9483), and then used this relationship to predict the total length of UWBM 95770 (with 95% confidence intervals) based on its proximal width alone. This method predicts UWBM 95770 to be 1167 ± 63 mm in total length, which suggests that UWBM 95770 represents a theropod similar in size to *Albertosaurus*, *Daspletosaurus*, or *Acrocanthosaurus* [63] and somewhat smaller than *Tyrannosaurus*.

## Discussion

### Large Theropods of the Late Cretaceous

Given what is preserved in UWBM 95770, a more specific taxonomic identification (i.e., beyond non-paravian theropod) is not possible on anatomical grounds. However, several candidate theropod subclades are plausible. For example, carcharodontosaurian allosauroids are known from the Cretaceous of North America, including the carcharodontosaurid *Acrocanthosaurus atokensis* from the Aptian-Albian [63, 64]. Other carcharodontosaurids are known from the Cenomanian of South America [65, 66] and Turonian of Asia [67] and both continents experienced faunal exchange with Laramidia throughout the Cretaceous [68], including hadrosaurid sister taxa spread between Laramidia and South America in the Campanian [69–71]. Neovenatorid carcharodontosaurians are also known from the Cenomanian through Santonian of South America [72–75], and importantly a new neovenatorid was recently described from the Cenomanian of Laramidia [76], but in rocks approximately 18 MY older than those of the Cedar District Formation. If UWBM 95770 pertains to either a carcharodontosaurid or neovenatorid carcharodontosaurian, it would represent the youngest known material belonging to that clade from North America.

Spinosaurid megalosauroids are known mostly from the Early Cretaceous and have not been found in North America [77, 78]. Megalosaurid megalosauroids are well known from the Late Jurassic of North America, but absent from the Cretaceous worldwide, with the tantalizing possible exception of a distal tibia from the Coniacian-Santonian Hidden Lake Formation of Ross Island, Antarctica [53]. Late Cretaceous abelisauroid ceratosaurians were very diverse and known mostly from the continents of Gondwana, although more recent finds have been made in Europe [73, 79, 80]. Both large and small-bodied abelisauroids share low, subtle fourth trochanters, which are distinct from the prominent ridge-like morphology seen in UWBM 95770 [53, 54]. Ceratosaurians and megalosauroids both retain a degree of anteromedial angling of the femoral head relative to the medio-lateral axis of the diaphysis not evidenced in UWBM 95570 [53], and although circumstantial, provenance also suggests that UWBM 95770 belongs to neither a megalosauroid nor a ceratosaurian.

Three subclades of Maniraptoriformes contain lineages known to have been capable of producing a femur the size of UWBM 95770, though two can be reasonably discounted. Ornithomimosaurs are known predominantly from the Cretaceous of Asia, although taxa have been discovered in Europe and Africa as well [81, 82]. In North America isolated ornithomimosaur material has been found in the Aptian/Albian of both Laramidia (Cloverly Formation; [83]) and the eastern subcontinent Appalachia (Arundel Formation; [84]). Two genera of ornithomimid ornithomimosaurs are known from the Campanian and Maastrichtian of Laramidia, *Struthiomimus* and *Ornithomimus*, but are too small to have produced UWBM 95770 [85, 86]. Recently the first nearly complete skeletons of the gigantic ornithomimosaur, *Deinocheirus mirificus*, were discovered in the upper Campanian or lower Maastrichtian Nemegt Formation of Mongolia [87]. At this point, there is not enough evidence to rule out a large deinocheirid ornithomimosaur as a source for UWBM 95770. However, if UWBM 95770 represented a deinocheirid, it would be the first record of the clade in North America.

Therizinosaurs are known from the Barremian through Maastrichtian of Laramidia, but higher diversity within the group is known from the Early Cretaceous through Maastrichtian of Asia [88–91]. The femora of therizinosaurs, especially the large-bodied therizinosaurids, were oval in cross-section, relatively wider mediolaterally than anteroposteriorly, which is a well-known phenomenon associated with the biomechanics of large body size [56, 92]. The known fourth trochanters of large therizinosaurids, like *Erliansaurus* and *Neimongosaurus*, are low and poorly developed [93, 94], suggesting that UWBM 95770 does not pertain to a large therizinosaurid.

Several taxa of caenagnathid oviraptorosaurs are known from the Campanian and Maastrichtian of Laramidia along the Western Interior Seaway [95]. However, these taxa and their Asian relatives, including the enormous *Gigantoraptor erlianensis*, lack a raised fourth trochanter and therefore could not have produced UWBM 95770 [96, 97].

The Late Cretaceous of North America hosted the height of tyrannosauroid diversity, with potentially up to nine genera known from the Campanian and Maastrichtian of Laramidia alone [4, 98]. *Lythronax argestes* was recently described from the middle Campanian (between 80.6±0.15 MA and 79.9±0.3 MA) of Utah as the earliest occurring member of Tyrannosauridae, overlapping the time of Cedar District Formation deposition [4, 41]. Other Campanian tyrannosauroids from Laramidia include *Daspletosaurus torosus*, *Gorgosaurus libratus*, *Teratophoneus curriei*, and *Bistahieversor sealeyi* [99–102]. The diversity of tyrannosauroids and their widespread occurrence on Laramidia suggest that it would not be surprising if UWBM 95770 belonged to this clade. An earliest Campanian origin of tyrannosaurids on Laramidia was proposed by Loewen et al. [4]. If the Baja-British Columbia (BBC) hypothesis is correct and UWBM 95770 represents a tyrannosauroid, then it would represent a larger individual than previously known from the mid-Campanian of southern Laramidia (see size estimation, above). On the other hand, if BBC is incorrect, then UWBM 95770 would be about 2 MY older than the oldest tyrannosauroid from northern Laramidia.

### Dinosaur Assemblages from the West Coast of Laramidia

In contrast to the rich fossil record of the eastern flank of Laramidia, the west coast of the subcontinent has yielded comparatively fragmentary material, with most occurrences based on specimens preserved in marine or near-marine environments [36]. Baja California, México has produced the largest diversity of dinosaurs from this region, but most of the taxa are based on isolated elements recovered from beach or nearshore lagoon and playa deposits [22, 27, 28, 103]. Isolated hadrosaurid bones (Hadrosauridae indet.) have been found in three successive formations in the area around El Rosario, with the majority coming from the middle ‘El Gallo’ Formation. On the basis of tall neural spines on the caudal vertebrae and a boot on the distal ischium, Morris [27] referred hadrosaur material from Baja California to *Hypacrosaurus altispinus*. Morris eventually revised the taxonomy of the Baja material to *cf*. *Lambeosaurus* before erecting a new species, *Lambeosaurus laticaudus* in 1981 [29, 35]. Recently, Prieto-Márquez et al. [31] conducted an in-depth analysis, including a critical evaluation of the postcrania, and assigned the Baja hadrosaurid bones to a new genus, *Magnapaulia*. *Magnapaulia laticaudus* was an enormous hadrosaurid, with an estimated body length in the realm of 12–15 meters [29, 31]. Other ornithischian remains from the ‘El Gallo’ include an isolated tooth referable to an indeterminate nodosaurid ankylosaurian [104], and indeterminate ceratopsian teeth [6, 27] that warrant full description. The saurischian record of the ‘El Gallo’ consists almost completely of isolated teeth, belonging to putative tyrannosaurids, the dromaeosaurid *Saurornitholestes sp*., and the troodontid *Troodon formosus* [32, 105]. Postcranial remains attributable to Tyrannosauridae [30] and a possible ornithomimid [105] are also known, although the ornithomimid material has not been figured or formally described, as far as we are aware.

Two theropod genera have been named from the underlying lower-middle Campanian La Bocana Roja Formation of Baja California, the enantiornthine bird *Alexornis antecedens* [33] and the enigmatic, large-bodied *Labocania anomala* [34]. *Labocania* is known exclusively from fragmentary cranial remains, parts of an ischium and pubis, a metatarsal II, and a chevron belonging to a single individual. It shares traits with tyrannosaurids, as well as with other large theropods [30, 106, 107]. Molnar [34] compared the cranial material of *Labocania* favorably with taxa now considered to be abelisaurid and neovenatorid theropods. *Labocania* warrants further study, especially with UWBM 95770 representing another large theropod from roughly coeval rocks.

Fossils from California (i.e., the Point Loma, Ladd/Williams, Moreno, Chico, and Budden Canyon formations) come from both fluvial and marine rocks, with the ankylosaur *Aletopelta coombsi* of the Point Loma Formation representing the rare instance of a near-complete unit of dermal ossifications, held together by skin [25]. Fragmentary hadrosaurid bones (Hadrosauridae indet.) are the most common dinosaur fossils throughout California. Two partial skeletons from the Moreno Formation were ascribed to the hadrosaur *Saurolophus* by Morris [22], but Prieto-Márquez et al. [23] later referred the skeletons and additional cranial material to the novel genus and species *Augustynolophus morrisi* (see also [108]). Turbidite facies within the marine Chico Formation have yielded isolated bones of three avian dinosaurs along with mollusk shells and teeth belonging to actinopterygians and sharks. Two of the avian bones pertain to toothed ornithurine birds (*Ichthyornis sp*. and *Hesperornis sp*.) common on the other side of Laramidia in the Western Interior Seaway, whereas the third is the ulna of an indeterminate neognath [19, 20]. In northern California the Lower Cretaceous marine Budden Canyon Formation has produced the stratigraphically lowest Pacific coast dinosaur fossil: a ‘hypsilophodontid’ hind limb (Ornithopoda indet.) [21]. Beyond the rare occurrence of dinosaurs, the Mesozoic marine rocks of California have routinely produced fossils of marine reptiles like thalattosaurs, ichthyosaurs, plesiosaurs, mosasaurs, and sea turtles as well as occasional fragmentary pterosaurs [19].

The sole Mesozoic dinosaur fossil known from Oregon is a sacrum that probably belongs to an indeterminate hadrosaurid. The specimen was discovered in the Cape Sebastian Sandstone Formation and, despite being mentioned in the literature [2, 18], it does not appear to have been accessioned at a public repository or to have been formally described.

UWBM 95770 belongs to a large theropod, likely that of a tyrannosaur, and is the sole Mesozoic dinosaur fossil from Washington, but not the first from the Nanaimo Group as both birds and a possible ornithomimid have been recovered from the Gulf Islands of British Columbia. Upper Cretaceous marine rocks of the Northumberland Formation on Hornsby Island, British Columbia have produced several limb elements of ornithurine and enantiornithine birds [16, 17]. There is evidence for at least two taxa of each clade, including species of relatively large body size for Mesozoic birds, which indicate a previously unknown diversity of birds in Pacific marine ecosystems. Bullard [109] described a weathered mid-caudal vertebra from the Cedar District Formation of Denman Island and referred it to? Ornithomimidae on the basis of a ventral keel on the centrum, amphiplatan articular faces, and comparisons with ornithomimid vertebrae at the Royal Tyrrell Museum of Palaeontology. The specimen is housed in the Royal British Columbia Museum (RBCM.EH2010.001.0001.01) and warrants restudy. Santonian-aged rocks from British Columbia have produced marine-adapted species including mosasaurs, an elasmosaurid, and desmatochelyid turtles [110]. A previously reported theropod tooth from Vancouver Island is now considered to be that of a mosasaur, *Kourisodon puntledgensis* [16, 110–112].

Two ornithischians have been found in marine strata of the Matanuska Formation of south-central Alaska. Gangloff [13] first reported a partial skull of the nodosaurid *Edmontonia sp*. from the Campanian-Maastrichtian Member 3 of the Matanuska Formation as the first Alaskan dinosaur outside of the North Slope. Subsequently, Pasch and May [14] described a relatively early occurring indeterminate hadrosaur from Member 4, which is well dated to the Turonian.
